# Use of outpatient healthcare services before and after the onset of unemployment: A register-based propensity score matched study from Finland

**DOI:** 10.1371/journal.pone.0288423

**Published:** 2023-08-09

**Authors:** Hanna Rinne, Jenni Blomgren

**Affiliations:** The Social Insurance Institution of Finland, Helsinki, Finland; Federal Teaching Hospital, Ido-Ekiti, NIGERIA

## Abstract

**Aims:**

The aim was to examine the use of outpatient healthcare services in different sectors of healthcare before and after the onset of unemployment and to study whether job loss affected the use of these services.

**Methods:**

Longitudinal individual-level register-based data was utilized on all individuals living in the City of Oulu, Finland, who became unemployed in 2017 (N = 1,999), their propensity matched controls (N = 1,999), and unmatched controls (N = 58,459) in a quasi-experimental design. Use of outpatient healthcare services was examined in one-month periods from 12 months before to 12 months after the onset of unemployment. Several socio-demographic factors, along with sickness and employment histories, were used for matching. Difference-in-differences analysis was used to measure the differences in the use of outpatient healthcare services between the unemployed and their matched controls.

**Results:**

The use of health services decreased significantly after the onset of unemployment. This was due to a decrease in the use of occupational health services. No change related to job loss was observed in the use of public or private healthcare services. The number of healthcare visits increased again after the unemployment ended. Difference-in-differences analyses showed that compared to propensity score matched controls, becoming unemployed reduced the use of health services.

**Conclusions:**

When access to occupational healthcare services ceases, other health services do not appear to fill the gap among those who become unemployed. Adequate healthcare services should be guaranteed to all population groups equally based on need, irrespective of the labour market status.

## Introduction

Many studies have shown that the unemployed have weaker physical and mental health compared to the employed population. This is due to both selection of those with poor health into unemployment and because unemployment may have adverse health effects [1–5]. Transition to unemployment can adversely affect living conditions and increase health risks through loss of income, social networks, and healthcare coverage as well as changes in health behaviour and coping styles [6]. Adverse health outcomes have been shown to increase with the duration of unemployment [1, 7]. These factors are also related to the use of health services [8].

Some studies have found that the unemployed use more healthcare services than the employed [9, 10], while other studies have shown that the unemployed use less services [1, 11–13] or that the use of services is at a similar level [14] compared to employed persons. Furthermore, results concerning the associations with the duration of unemployment have been conflicting [12, 15, 16]. Previous studies have mostly examined the associations of unemployment with the use of healthcare services. There are also some recent studies on the actual effect of job loss on the use of healthcare services, controlling for fixed effects. A German study found no effect between the onset of unemployment and the use of healthcare services [2], whereas studies from the US found some decrease in the use of healthcare services [17] and an increase in the use of mental health services [18].

The effect of lob loss on the use of healthcare services depends on the country’s health insurance system [19]. In principle, Finland has a universal healthcare system. Public healthcare is accessible to everyone, but waiting times may be long and there is usually a moderate client fee (in 2018, 20.60 euros per visit at the public healthcare centre and 41.20 per visit at the specialised healthcare [20]). Employed persons can use occupational healthcare services, which are quickly accessible and free of charge at the point of delivery. Private healthcare services are available to those who can afford them. [21] The volume of private health insurance has increased but its role is still minor, because public healthcare is available for everyone and employees are covered by occupational healthcare. In 2017, only 16% of adults had private health insurance [22]. Private health insurance may increase the use of private health services, but research on this is lacking in Finland. According to previous Finnish studies [12–14, 23], employed persons mostly use occupational healthcare services, whereas the unemployed mostly use public healthcare services. Furthermore, the use of private healthcare services is more common among the employed than among the unemployed.

However, while there are several studies comparing the use of healthcare services by occupational status, including the unemployed, there is a lack of knowledge on what actually happens to the use of healthcare services after the onset of unemployment. On the one hand, the onset of unemployment may entail adverse health effects and, thus, increase the need for healthcare services. On the other hand, in Finland, the onset of unemployment entails loss of access to the free-of-charge occupational healthcare services, which may as such decrease the overall use of healthcare services. To complement previous knowledge on the associations, more information is needed on the effects of unemployment on the use of healthcare services.

## Aims

The aim of the study was to examine the use of outpatient healthcare services before and after the onset of unemployment. In particular, we aimed to measure the development in the use of healthcare services in different sectors of healthcare and to identify potential differences in the use of healthcare services by the length of unemployment. Furthermore, we examined whether unemployment had an effect on the use of healthcare services.

## Materials and methods

### Study population

We used a large individual-level register-based data set on all inhabitants living in the City of Oulu, Finland, in years 2013–2018 [24]. Register data were retrieved from the Social Insurance Institution of Finland, the municipality of Oulu, the National Institute for Health and Welfare, four largest occupational healthcare providers in Oulu, the Finnish Centre for Pensions, Statistics Finland, and Finnish Tax Administration. Among the persons included in the data set, we identified 18–59-year-old persons who became unemployed during 2017, had not been unemployed during the preceding three months prior to the onset of the first unemployment spell in 2017, and whose unemployment spell lasted for at least 30 days (N = 10,562).

**Fig 1:**
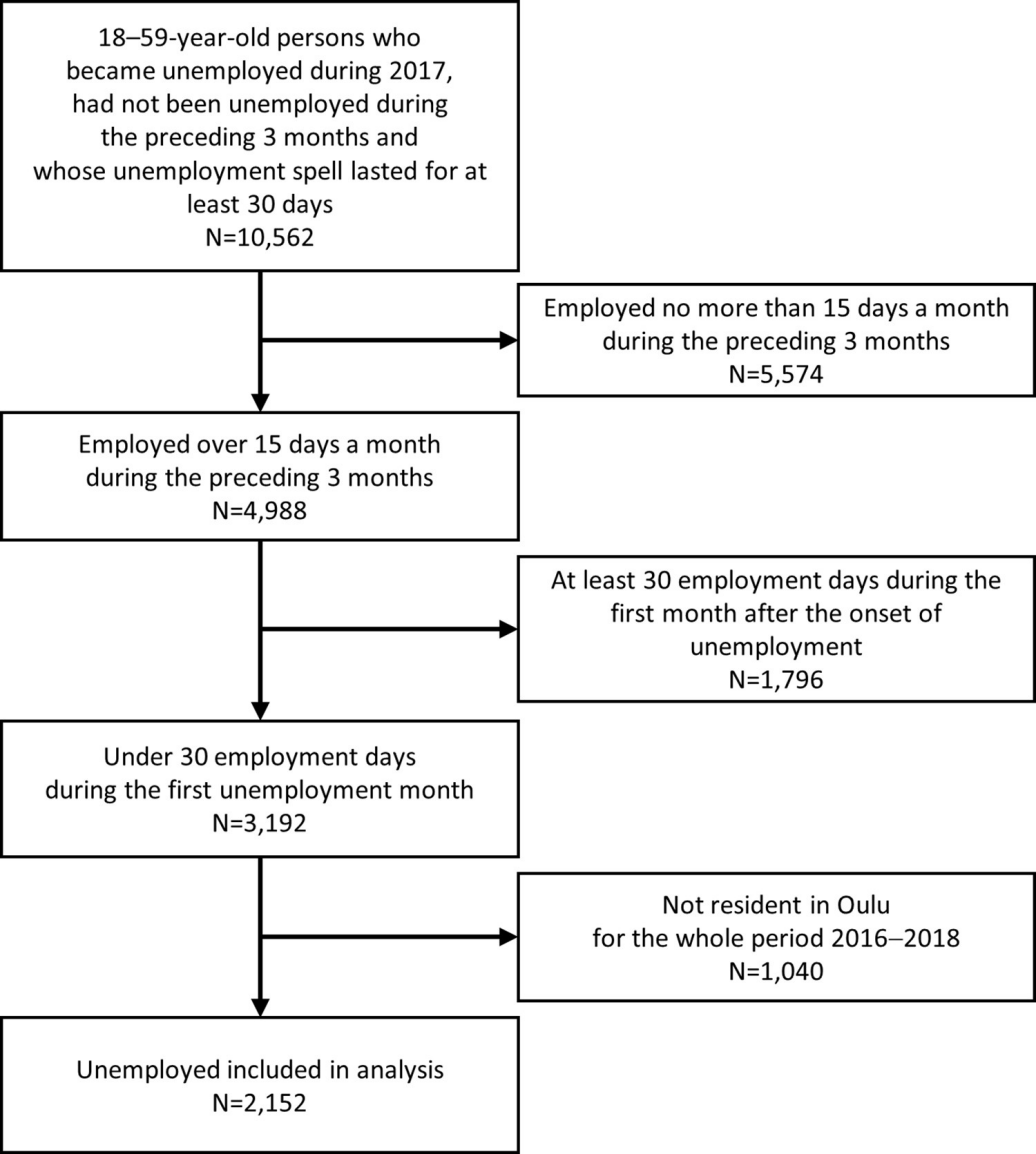
Flowchart of the study population.

To include persons whose unemployment spell started after a period of employment, the analyses were further restricted to those who had been employed for over 15 days per month during the preceding three months prior to the onset of the index unemployment spell (N = 4,988). In order to exclude persons receiving adjusted unemployment allowance during part-time work, those who had at least 30 employment days during the first month after the onset of unemployment were not included. Finally, due to the measurement of dependent variables of the use of healthcare services (see below), the analyses were restricted to persons who were residents in Oulu in all years 2016–2018. This left us with a dataset that included 2,152 unemployed persons (Fig 1).

### Use of healthcare services and the follow-up setting

We calculated the number of visit days to a doctor or a nurse in outpatient healthcare services over one-month periods: 12 months before the start of the index unemployment spell and 12 months after the start of the spell. We included face-to-face visits, excluding visits related to oral healthcare services. Because it was not possible to count the number of visits during the same day in all data sets reliably, we calculated the number of attendance days for each subject as a proxy for the number of visits.

Outpatient healthcare services included

- public primary healthcare services from the registers of the city of Oulu,
- occupational healthcare services from the registers of the four largest occupational healthcare providers,
- private healthcare services from the registers of the Social Insurance Institution of Finland,
- public specialised healthcare from the registers of the Finnish Institute for Health and Welfare (Care Register for Health Care Hilmo), and
- emergency visits from the registers of the City of Oulu and Hilmo.

Visits to healthcare services in different sectors of healthcare were examined both separately and as a whole.

### Background factors

We measured sex and age (classified to five-year age groups) at the end of year 2016. Education (tertiary, secondary, primary), occupational class (employed, unemployed, student, other or missing information on occupational class), and marital status (married, unmarried or missing information of marital status, divorced/widowed) were measured at the end of year 2016, and income quintiles of gross yearly taxable income were measured from year 2016 (the income of those with no registered taxable income was set to zero). Information was obtained from the registers of the Social Insurance Institution of Finland, Statistics Finland and Finnish Tax Administration (S1 Table).

To adjust for employment, unemployment, and sickness absence history of the study population, recorded days in each of these states were calculated for 12 individual months before the start of the index unemployment spell. The monthly variables were set to 1 if the number of days in each of the three states were at least 15, and to 0 otherwise. The number of chronic diseases and previous use of healthcare services were included to adjust for previous health status. Chronic diseases (yes/no during the 12 months prior to the index spell) were measured through special reimbursements of medicines used in the treatment of severe and long-term diseases [25]. Use of inpatient healthcare services during the 12 months prior to the index spell was used as a dummy variable (yes/no). Information on the employment factors were obtained from the registers of the Finnish Centre for Pensions and health-related factors from the Social Insurance Institution of Finland and the National Institute for Health and Welfare.

### Propensity score matching

The study utilised a quasi-experimental design with propensity-score matched controls and difference-in-differences analysis. We used propensity score matching to define matched controls for the unemployed study population [26].

The matched controls were selected from the population who did not become unemployed but otherwise met the same criteria as the intervention group: aged 18–59 at the end of year 2016, employed over 15 days per month during the preceding three months, and residing in Oulu for the whole period 2016–2018 (N = 60,458). For the control group, we randomised the index day to be any date in year 2017.

We estimated propensity scores using logistic regression model with treatment assignment (not unemployed vs. unemployed) as the dependent variable and used nearest neighbour matching 1:1 with a calliper width of 0.025. In matching, we used the above-mentioned background factors and use of any outpatient healthcare services during every 12 months before unemployment. To examine the balance diagnostic after the propensity score matching, we compared the prevalence and means of background factors between the unemployed and their controls with the standardised difference. [26].

In propensity score matching, a matched control was found for almost all of the persons in the unemployed group. In total, 153 unemployed persons (i.e. 7% of all unemployed) were not matched and were therefore excluded from the analysis. Compared to the matched unemployed, those who were unmatched and thus excluded, had more unemployment days during the preceding 12 months, were more often married and had lower income. There was no difference in the use of healthcare services during the follow-up. The remaining number of unemployed was 1,999, as was the number of their matched controls. The rest of the control population remained as unmatched controls (N = 58,459).

The background factors of the unemployed group, their matched controls, and unmatched controls are described in detail in S1 Table and in condensed form in Table 1. The distribution of background factors was more similar between the unemployed and their matched controls than between the unemployed and their unmatched controls. All variables reached the required level of similarity (the standardised difference of the mean being <10%) (S2 Table).

**Table 1:** Distribution of background factors (%) among the unemployed, their matched controls, and unmatched controls.

Variable	Unemployed	Matched controls	Unmatched controls
Sex in 2016			
Men	46	47	52
Women	54	53	48
Age in 2016			
18–29	32	34	21
30–39	30	29	29
40–49	22	22	28
50–59	17	15	23
Education			
Tertiary	41	39	53
Secondary	50	52	41
Primary	9	8	6
Occupational class in 2016			
Employed	88	87	94
Unemployed	6	6	0
Student	3	3	3
Other	3	3	3
Income quintiles in 2016			
1^st^ quintile (lowest)	41	43	18
2^nd^ quintile	28	28	19
3^rd^ quintile	15	14	20
4^th^ quintile	11	10	21
5^th^ quintile (highest)	5	5	21
Marital status in 2016			
Married	37	34	50
Unmarried	52	56	39
Divorced/widow	11	10	11
Over 15 employment days during the 6^th^ month before unemployment	91	90	98
Over 15 unemployment days during the 6^th^ month before unemployment	4.9	5.1	0.2
Over 15 sickness absence days during the 6^th^ month before unemployment	2.1	1.6	1.5
Chronic diseases in 2016	17	17	17
Inpatient care in 2016	6	6	6
Number of visits in outpatient healthcare services during the 6^th^ month before unemployment			
0 visit	78	78	80
1 visit	15	16	14
2+ visits	7	6	6
**Total**	100	100	100
**N**	1,999	1,999	58,459

### Analysis

In the descriptive analysis, we examined the average monthly number of visits in outpatient healthcare services 12 months before and 12 months after the onset of unemployment. We analysed the use of each outpatient healthcare service type separately and all together. We also examined the use of any outpatient healthcare services by the duration of unemployment. It was categorised to three groups: no more than 3 months, 3–9 months, and over 9 months.

After matching, we used difference-in-differences (DiD) analysis [27] to measure the differences in the use of outpatient healthcare services between the unemployed and their matched controls. We modelled the number of outpatient visits with a regression model and compared the average number of visits in one-month and three-month periods before and after the onset of unemployment. We did not adjust any background factors in the regression models, because the study population was successfully matched with controls (covariate balance was achieved; standardised difference of the mean <10%) [28]. Stata 14.2 was used for analysis.

### Ethics statement

As the study was based on register data and there was no contact with the subjects, no ethical preliminary assessment was required under Finnish law. The data used in the study were fully pseudonymised before the access and no informed consent was required. The data were accessed through permissions from the City of Oulu, the Social Insurance Institution of Finland, National Institute for Health and Welfare, the Finnish Center for Pensions, four large occupational health care providers, Statistics Finland and the Finnish Tax Administration. The legal restrictions prevent from sharing data publicly since use of sensitive individual-level health data is strictly regulated by law in Finland. Permission to access the data can be applied from the register holders.

## Results

### Distribution of background factors

Compared to the unmatched controls, the unemployed and their matched controls were more likely to be women, younger, unmarried, and not employed at the end of the preceding year. In addition, they were more likely to have a lower education level and lower income (Table 1). The unemployed had less employment days and more unemployment days before the index day. However, sickness absence days, chronic diseases, and disability pension were equally prevalent.

The length of the unemployment period varied between 30 and 730 days. The mean length was 211 days (SD 195). The length of unemployment was no more than 3 months for 41%, 3–9 months for 30%, and over 9 months for 29% of the unemployed study population.

### Use of different healthcare services among the unemployed and their matched and unmatched controls

**Fig 2:**
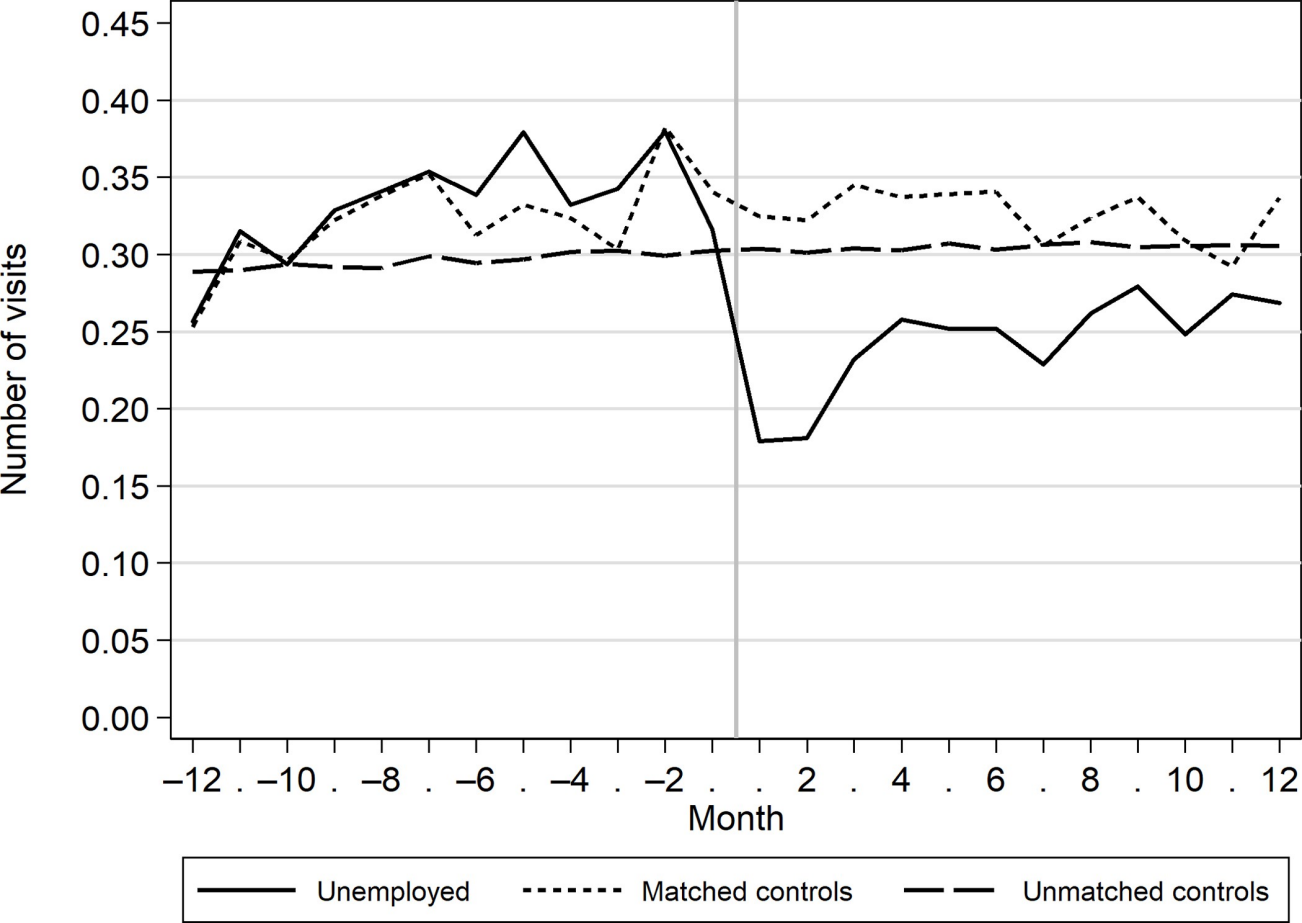
The monthly number of visits to outpatient healthcare services among the unemployed and their matched and unmatched controls 12 months before and 12 months after the onset of unemployment (unemployed) or the index date (matched controls).

Among the unemployed, the number of visits to healthcare services increased during the year before the onset of unemployment (Fig 2). The numbers dropped drastically right after unemployment began but rapidly started to increase again. After 12 months, the number of visits was at same level as 12 months before the start of unemployment.

Among matched controls, the use of healthcare services remained at a higher level throughout the follow-up period. Among the unmatched controls, use of healthcare services remained at a steady level both before and after the index date.

**Fig 3:**
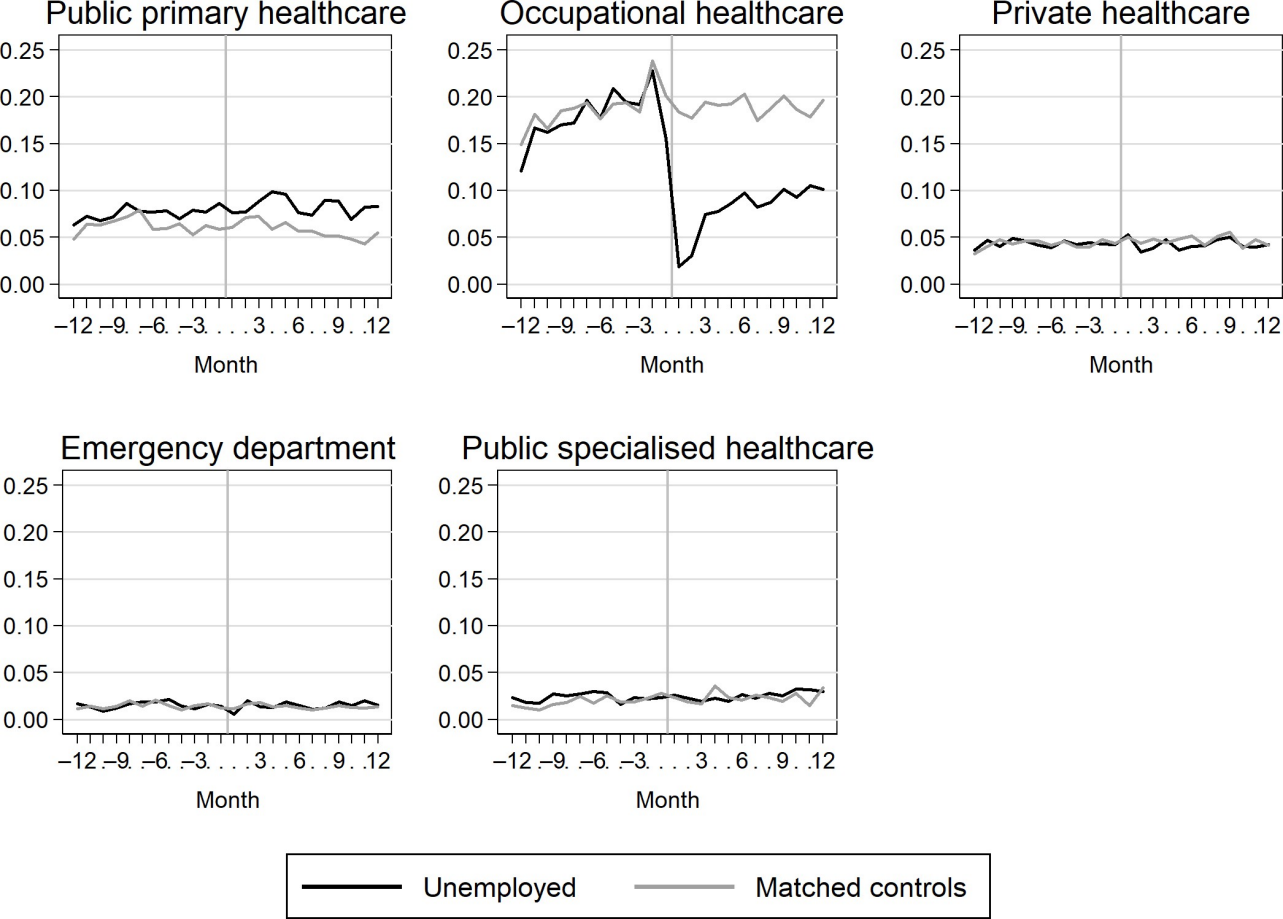
Monthly number of visits to outpatient healthcare services in different sectors of healthcare among the unemployed and their matched controls 12 months before and after the onset of unemployment (unemployed) or the index date (matched controls).

The biggest changes occurred in the use of occupational healthcare services (Fig 3). The monthly number of visit days among those who became unemployed dropped from 0.16 to 0.02 when comparing the situation 1 month before and 1 month after the onset of unemployment. After that, the number of visits increased again but did not reach the same level as before unemployment.

The use of public primary healthcare services was at a slightly higher level among the unemployed than among the controls after the first quarter following the onset of unemployment. The use of other services remained unchanged among the unemployed, i.e. at the same lower level as among the controls.

**Fig 4:**
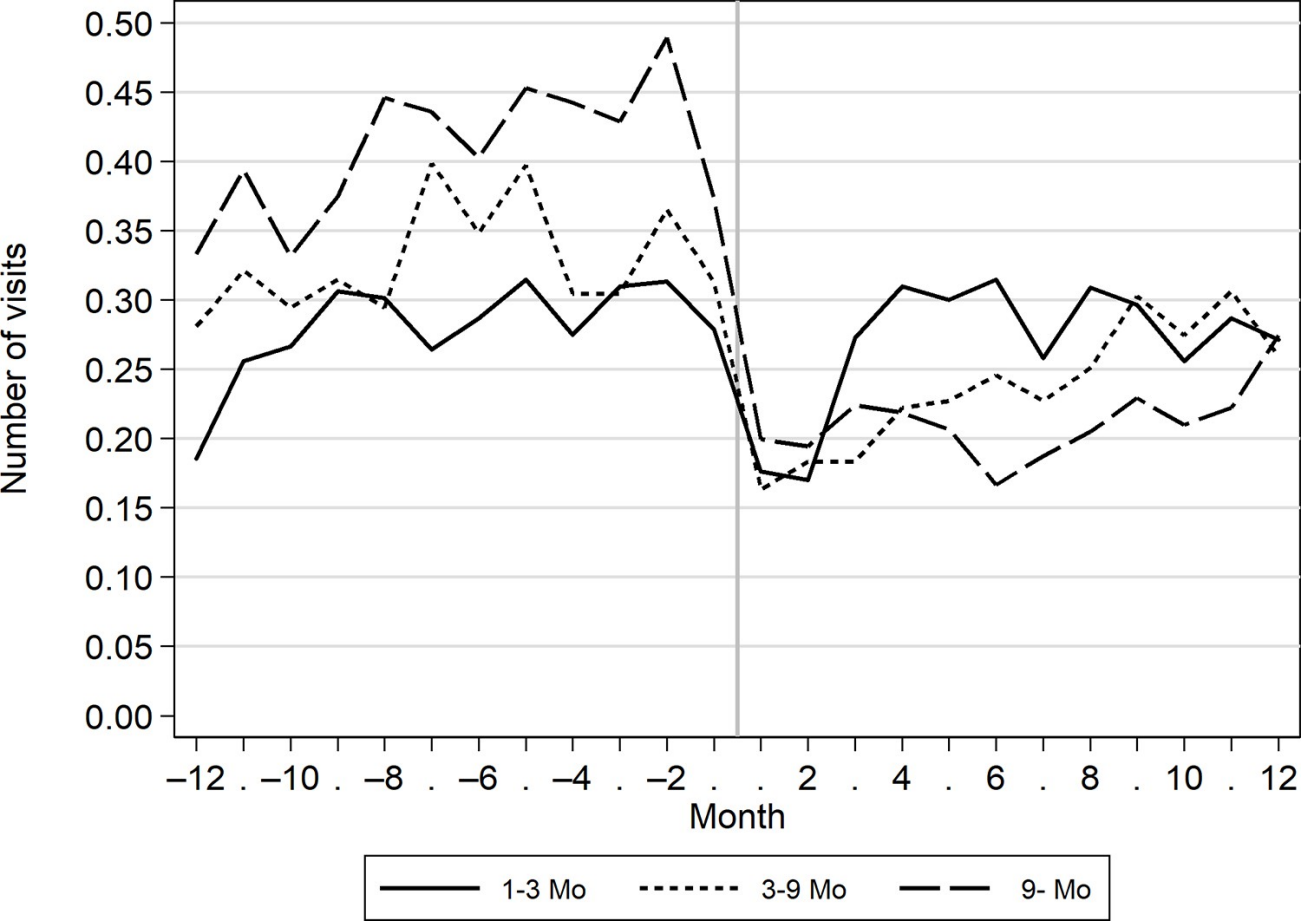
Monthly number of visits to outpatient healthcare services among the unemployed 12 months before and after the onset of unemployment by the duration of unemployment.

Before the onset of unemployment, those whose unemployment spell lasted no more than 3 months used healthcare services less frequently than those whose unemployment lasted over 9 months (Fig 4). After the onset, the results were reversed because the number of healthcare visits increased after the end of the unemployment spell.

Comparing the number of visits during the first month before and first month after the onset of unemployment, the average number of visits to any outpatient healthcare services decreased among the unemployed but remained at the same level among their matched controls (DiD: -0.12, 95% CI: -0.19 –-0.05) (Table 2). The decrease was due to changes in occupational healthcare services (DiD -0.12, 95% CI: -0.16 –-0.08), and no significant difference was observed for other services. The results were similar when using three-month observation periods but with larger DiD estimates.

**Table 2:** Average number of visits in outpatient healthcare among the unemployed (N = 1,999) before and after the onset of unemployment and among their matched controls (N = 1,999) before and after the index day, the difference between these periods, and the difference in these differences (DiD) with 95% confidence intervals.

	Unemployed			Matched controls				
	Before	After	Diff	Before	After	Diff	DiD	95% CI
**1-month period**								
Any outpatient care	0.32	0.18	**-0.14**	0.34	0.33	-0.02	**-0.12**	(-0.19;-0.05)
Public primary healthcare	0.09	0.08	-0.01	0.06	0.06	0.01	-0.01	(-0.04;-0.02)
Occupational healthcare	0.16	0.02	**-0.14**	0.20	0.18	-0.02	**-0.12**	(-0.16;-0.08)
Private healthcare	0.04	0.05	0.01	0.04	0.05	0.01	0.01	(-0.02;0.03)
Emergency department	0.01	0.01	-0.01	0.01	0.01	0.00	-0.01	(-0.02;0.00)
Public specialised healthcare	0.02	0.03	0.00	0.03	0.02	-0.01	0.01	(-0.02;0.04)
**3-month period**								
Any outpatient care	1.04	0.59	**-0.45**	1.03	0.99	-0.03	**-0.41**	(-0.57;-0.26)
Public primary healthcare	0.24	0.24	0.00	0.17	0.20	0.03	-0.03	(-0.09;0.03)
Occupational healthcare	0.56	0.12	**-0.43**	0.62	0.58	-0.05	**-0.38**	(-0.49;-0.28)
Private healthcare	0.13	0.13	0.00	0.13	0.14	0.01	-0.02	(-0.07;0.04)
Emergency department	0.04	0.04	0.00	0.04	0.05	0.00	-0.01	(-0.03;0.02)
Public specialised healthcare	0.07	0.07	0.00	0.07	0.06	-0.01	0.01	(-0.05;0.09)

## Discussion

### Main results

This study analysed the use of outpatient healthcare services before and after the onset of unemployment, utilising a quasi-experimental design with propensity-score matched controls and difference-in-differences analysis. The results showed that the use of health services decreased significantly after the onset of unemployment due to a decrease in the use of occupational health services. The number of healthcare visits increased again after unemployment ended. Compared to matched controls, becoming unemployed seems to reduce the use of health services.

### Interpretation of the results

The results of this study are in line with the results of a US study [17] that found that those who lost their health insurance coverage due to job loss as well as those with chronic diseases reduced their use of healthcare services after losing their jobs. Other US studies have also found that, compared to the employed, those who become unemployed are more likely to report lack of healthcare coverage and to delay healthcare services due to cost [1, 7]. On the other hand, in contrast to our results, a German study found that the number of doctor’s visits was not associated with the onset of unemployment [2], and another study from the US [18] reported an increase in family mental health spending after job loss.

Differences in the healthcare systems may explain at least some of the differences obtained from different countries. In Finland, the impact of the health services system is strong, despite the universal healthcare. Occupational healthcare is offered to those employed, while non-employed groups mostly rely on public services [12–14, 23]. Barriers of use are low in occupational healthcare: The services are rather easily accessible and free of charge for the user at the point of delivery, and gatekeeping is low. Under certain conditions, also the dismissed employees have the right to occupational healthcare for another six months after the start of unemployment. However, the unemployed rely mostly on public healthcare, where gatekeeping may be strong and provision of services more limited than in occupational healthcare [12–14, 23].

One explanation for the decreased use of healthcare services after the onset of unemployment in our data may be that the unemployed do not have to certify their illness and incapacity for work to the employer. Work-related health checks and the need for sickness absence certificates may increase the use of health services among employed persons. This is also indicated by the results concerning the duration of unemployment. Re-employment is more likely for healthier individuals [2]. Therefore, the increased number of visits after unemployment for those with shorter unemployment spells may partly be due to work-related health checks or sickness absence certificates for the employer. However, our data did not allow for a detailed examination of the reasons for healthcare appointments to explore this explanation further. More research is needed on the role of sickness absence certification and its effects on the increasing use of healthcare services among the employed population compared to the unemployed.

Among those who remained unemployed for a longer period of time, the use of health services was common before job loss but after becoming unemployed, the use remained at a lower level. This indicates a change in the availability of services. In addition, the above-mentioned effect of not needing sickness absence certificates during unemployment may explain part of this result. Among the long-term unemployed, the health services have been found to be inadequate [12] and their use polarised: Some use services frequently, others do not use them at all [15–16]. Overall, the unemployed have a higher risk for unmet healthcare needs [29, 30] compared to the employed.

Loss of occupational healthcare may have long-lasting effects on the health of those who become unemployed. Long-term illnesses are often treated in occupational healthcare. When access to occupational healthcare ends, the treatment relationship is also terminated [31]. If a new care relationship is not established at public services, many health conditions may remain untreated. Due to the underuse of healthcare services, diseases may not be recognised in time, which may cause more serious long-term consequences for health. Underuse of healthcare services among the unemployed has a weakening effect not only on the health of the unemployed but also on the identification of impaired work capacity [32, 33].

In Finland, public healthcare providers are responsible for organising health checks for the unemployed, and employment services are responsible for guiding the unemployed to the health checks. Despite the responsibilities of the authorities, these health checks are not carried out nearly as often as expected [33]. In order to be competent to steer the unemployed to health checks and, when necessary, to other services immediately after the onset of unemployment, employment services needs sufficient expertise in identifying the need for health services among the unemployed.

### Strengths and limitations

The main strength of our study was the possibility to use a longitudinal individual-level register-based data set that includes all inhabitants of the City of Oulu, Finland. Register data are considered reliable, and there is no self-report bias and very little missing information. Our data comprehensively covered all outpatient health services; including occupational healthcare, which is rarely available in registers. The data also included a wide variety of background factors that allowed for quasi-experimental methods. Our study also had limitations. Our data did not include information on the reasons or diagnoses behind the healthcare visits. Furthermore, the data included inhabitants of only one Finnish city, and the results may thus not be generalizable to other regions. Oulu is the fifth largest city in Finland. The availability of health services is more comprehensive in large cities than in smaller towns. Furthermore, compared to the entire population of Finland, residents of Oulu are slightly younger and the level of education as well as the unemployment rate are higher [22]. Finally, our results are highly dependent on the Finnish healthcare system and may not be generalizable to other countries with different systems.

We limited our study to outpatient visits, because we wanted to measure the development in the use of outpatient healthcare services in different sectors of healthcare that constitute the base of the Finnish healthcare system. Future studies should strive to analyze also other types of care, such as use of inpatient care and rehabilitation before and after the onset on unemployment.

## Conclusions

The overall use of outpatient health services decreases significantly after job loss in Finland because of a decrease in the use of occupational health services. When access to occupational healthcare ceases, other health services do not seem to fill the gap. Adequate and easily accessible healthcare services should be guaranteed to all population groups equally based on need, irrespective of the labour market status or other socio-demographic factors.

## Supporting information

S1 Table Background variables, their classifications and sources. (PDF)

S2 Table Distribution of background factors (%) among the unemployed, their matched controls, and unmatched controls and standardised difference of the mean between the unemployed and their matched controls. (PDF)
