# Supplementary material for: Use of outpatient healthcare services before and after the onset of unemployment: A register-based propensity score matched study from Finland
Source: PLoS One. 2023 Aug 9;18(8):e0288423. doi: 10.1371/journal.pone.0288423 (PMC10411812; doi:10.1371/journal.pone.0288423)
Supplement: S1 Table — (PDF) [file pone.0288423.s001.pdf]

S1 Table. Background variables, their classifications and sources.

Variable	Classification	Source
Sex in 2016	Men, women	Social Insurance Institution of Finland
Age in 2016	18–29, 30–39, 40–49, 50–59	Social Insurance Institution of Finland
Education	Tertiary, secondary, primary	Statistics Finland
Occupational class in 2016	Employed, unemployed, student, other	Statistics Finland
Income in 2016	Quintiles	Finnish Tax Administration
Marital status in 2016	Married, unmarried, divorced/widow	Statistics Finland
Employment days during the 1–12th month before unemployment	Under 15 days, over 15 days	Finnish Centre for Pensions
Unemployment days during the 1–12th month before unemployment	Under 15 days, over 15 days	Finnish Centre for Pensions
Sickness absence days during the 1–12th month before unemployment	Under 15 days, over 15 days	Social Insurance Institution of Finland
Chronic diseases in 2016	Yes, no	Social Insurance Institution of Finland
Inpatient care in 2016	Yes, no	National Institute for Health and Welfare
