# Supplementary material for: Use of outpatient healthcare services before and after the onset of unemployment: A register-based propensity score matched study from Finland
Source: PLoS One. 2023 Aug 9;18(8):e0288423. doi: 10.1371/journal.pone.0288423 (PMC10411812; doi:10.1371/journal.pone.0288423)
Supplement: S2 Table — (PDF) [file pone.0288423.s002.pdf]

S2 Table. Distribution of background factors (%) among the unemployed, their matched controls, and unmatched controls and standardised difference of the mean between the unemployed and their matched controls.

Variable	Unmatched controls	Matched controls	Unemployed	Standardised difference of the mean
**Sex in 2016**				
Men	52	47	46	-2.1
Women	48	53	54	2.1
**Age in 2016**				
18–19	2	2	3	1.7
20–24	8	14	13	-5.1
25–29	11	17	16	-3.4
30–34	13	15	16	0.6
35–39	15	13	14	1.3
40–44	14	11	11	-0.9
45–49	13	10	10	-0.3
50–54	14	9	10	3
55–59	10	6	7	4.5
**Education in 2016**				
Tertiary	53	39	41	3.9
Secondary	41	52	50	-4.5
Primary	6	8	9	1.2
**Occupational class in 2016**				
Employed	94	87	88	1.4
Unemployed	0	6	6	-1.4
Student	3	3	3	0
Other	3	3	3	-0.9
**Income quintiles in 2016**				
1st quintile (lowest)	18	43	41	-4.7
2nd quintile	19	28	28	-0.2
3rd quintile	20	14	15	1.4
4th quintile	21	10	11	4.4
5th quintile (highest)	21	5	5	0.2
**Marital status in 2016**				
Married	50	34	37	5.2
Unmarried	39	56	52	-6.7
Divorced/widow	11	10	11	2.4
**Over 15 employment days during the month before unemployment**				
–12 month	95	68	68	-0.3
–11 month	96	70	70	-0.3
–10 month	96	78	78	1.2
–9 month	96	81	82	1
–8 month	97	84	85	2.8
–7 month	97	86	88	5.1
–6 month	98	90	91	4.7
–5 month	98	94	93	-0.2
–4 month	99	97	97	0.3
–3 month	100	100	100	
–2 month	100	100	100	
–1 month	100	100	100	

**Over 15 unemployment days during the month before unemployment**				
–12 month	0.9	27.8	28.2	1.2
–11 month	0.6	25.6	26.0	1.1
–10 month	0.7	16.6	16.8	0.5
–9 month	0.6	13.5	13.3	-0.8
–8 month	0.5	10.6	10.4	-0.9
–7 month	0.3	8.6	8.5	-0.7
–6 month	0.2	5.1	4.9	-1.3
–5 month	0.1	3.2	3.5	1.9
–4 month	0.0	1.0	1.4	4.2
–3 month	0.0	0.0	0.0	
–2 month	0.0	0.0	0.0	
–1 month	0.0	0.0	0.0	
**Over 15 sickness absence days during the month before unemployment**				
–12 month	1.4	1.1	1.2	1.3
–11 month	1.4	1.4	1.5	0.4
–10 month	1.4	1.9	1.9	-0.4
–9 month	1.4	1.8	2.1	2.7
–8 month	1.4	2.1	2.0	-0.4
–7 month	1.5	1.7	2.1	3.1
–6 month	1.5	1.6	2.1	3.4
–5 month	1.5	1.7	2.1	3
–4 month	1.5	1.8	2.0	1.1
–3 month	1.5	1.7	2.1	3
–2 month	1.6	1.9	2.0	0.8
–1 month	1.6	1.6	1.9	2.7
**Chronic diseases**	17	17	17	1.3
**Inpatient care**	6	6	6	-0.2
**Use of outpatient healthcare during the month before unemployment**				
–12 month				
0 visit	80	83	83	-1
1 visit	14	12	12	2.7
2+ visits	6	5	5	-2.2
–11 month				
0 visit	81	78	79	1.9
1 visit	13	15	15	-2.2
2+ visits	6	6	6	0
–10 month				
0 visit	81	79	79	-0.4
1 visit	13	16	16	-1.4
2+ visits	6	5	6	2.8
–9 month				
0 visit	80	78	79	1.7
1 visit	14	16	15	-2.7
2+ visits	6	6	7	1
–8 month				
0 visit	81	78	79	1.1
1 visit	13	14	14	0
2+ visits	6	7	7	-1.8

–7 month				
0 visit	80	77	79	4.3
1 visit	14	15	14	-2.2
2+ visits	6	8	7	-4
–6 month				
0 visit	80	78	78	1.1
1 visit	14	16	15	-2.7
2+ visits	6	6	7	2.1
–5 month				
0 visit	80	78	77	-2.8
1 visit	14	14	15	1
2+ visits	6	7	8	3.1
–4 month				
0 visit	80	80	79	-1.7
1 visit	14	13	14	2.3
2+ visits	6	7	7	-0.4
–3 month				
0 visit	80	80	78	-4
1 visit	14	14	15	3.3
2+ visits	6	7	7	2
–2 month				
0 visit	80	76	76	0
1 visit	13	16	16	1.1
2+ visits	6	8	8	-1.6
–1 month				
0 visit	80	79	79	0.4
1 visit	14	13	14	2.9
2+ visits	6	8	7	-4.6
Total	100	100	100	
N	58,459	1,999	1,999	
